# Drone-Mountable Gas Sensing Platform Using Graphene Chemiresistors for Remote In-Field Monitoring

**DOI:** 10.3390/s22062383

**Published:** 2022-03-19

**Authors:** Jaewoo Park, Franklyn Jumu, Justin Power, Maxime Richard, Yomna Elsahli, Mohamad Ali Jarkas, Andy Ruan, Adina Luican-Mayer, Jean-Michel Ménard

**Affiliations:** Department of Physics, University of Ottawa, Ottawa, ON K1N 6N5, Canada; jpark4@uottawa.ca (J.P.); fjumu014@uottawa.ca (F.J.); jpowe074@uottawa.ca (J.P.); mrich154@uottawa.ca (M.R.); yelsa078@uottawa.ca (Y.E.); mjark066@uottawa.ca (M.A.J.); a2ruan@uwaterloo.ca (A.R.); luican-mayer@uottawa.ca (A.L.-M.)

**Keywords:** microcontroller, graphene gas sensor, drone mountable, in-field performances

## Abstract

We present the design, fabrication, and testing of a drone-mountable gas sensing platform for environmental monitoring applications. An array of graphene-based field-effect transistors in combination with commercial humidity and temperature sensors are used to relay information by wireless communication about the presence of airborne chemicals. We show that the design, based on an ESP32 microcontroller combined with a 32-bit analog-to-digital converter, can be used to achieve an electronic response similar, within a factor of two, to state-of-the-art laboratory monitoring equipment. The sensing platform is then mounted on a drone to conduct field tests, on the ground and in flight. During these tests, we demonstrate a one order of magnitude reduction in environmental noise by reducing contributions from humidity and temperature fluctuations, which are monitored in real-time with a commercial sensor integrated to the sensing platform. The sensing device is controlled by a mobile application and uses LoRaWAN, a low-power, wide-area networking protocol, for real-time data transmission to the cloud, compatible with Internet of Things (IoT) applications.

## 1. Introduction

Detection and identification of volatile compounds in remote locations for air quality monitoring are crucial to establish and enforce environmental policies on greenhouse emission or provide early warning for dangerous air-borne agents. To enable these applications, gas sensors must not only feature high sensitivity and selectivity to specific molecules, but also have practical in-field characteristics, such as being compact, light-weight, deployable, and low cost [1,2,3]. One strategy consists of miniaturizing the design of gas sensing technologies first developed in research in laboratories. This approach was used for gas chromatography/liquid chromatography-mass spectroscopy (GC/LC-MS) [4,5], and ion mobility spectroscopy (IMS) [6,7,8]. In these contexts, such an approach can ensure high sensitivity as well as selectivity, but the structural complexity of these devices puts a limit on their minimal size and cost.

Previous work demonstrated that, when exploring new compact sensing technologies, graphene-based chemiresistors satisfy many requirements for gas sensing applications [9,10]. For example, their lightweight, low power consumption, and chemical stability position them advantageously to play a key role in future sensing technologies [11,12,13,14,15]. Notably, graphene-based sensors have an especially high gas detection sensitivity that can be attributed to the two-dimensional nature of the material, ensuring that adsorbed molecules on the surface significantly affect its macroscopic electrical properties [16,17]. Scalable approaches to fabrication of graphene, for example by chemical vapor deposition (CVD) or liquid phase exfoliation (LPE), provide a pathway to large-scale fabrication of sensor arrays [18]. Those advantages combined with recent progress on improving selectivity of graphene-based chemiresistors have made possible to envision the large-scale deployment of this technology for environmental monitoring and warfare agents detection [10,19]. Finally, graphene-based sensors have demonstrated mechanical strength and flexibility, which are essential characteristics to many devices used in real-life applications [20].

In parallel to the development of smaller gas sensing devices, programmable 32-bit microcontrollers (μC), such as ESP32, are increasingly used to achieve compact and portable processing platforms [21,22,23,24]. In addition to their small size and low cost, they are energy efficient and can readily interface with electronic hardware and components, such as Wi-Fi and Bluetooth transmitters. These hardware features are critical to enable Internet of Things (IoT) applications and allow emerging technologies, such as in-field gas sensing, [21,22,25,26] to be integrated into the design of unmanned automated vehicles. Among those, drones offer the ability to sample large remote areas, including those otherwise inaccessible due to geographical landscape or safety concerns. Therefore, there is considerable interest in developing sensing platforms with an architecture compatible with drone sizes, shapes, and movements [27]. A specific challenge is ensuring that sensing devices can be operated over large distances for remote monitoring and provide accurate readings despite the drone’s rapid movement and environmental fluctuations. In particular, changes in humidity and temperature are known to induce changes in graphene’s electrical properties, potentially leading to a high noise level when graphene is used as a chemiresistor [28,29,30,31]. Recent research showed that environmentally induced signal variations can be resolved when the device is kept at a high temperature of 110 °C [27]. However, this technique requires a heat-resistant hardware configuration and demands a higher power consumption, which reduces the battery lifetime. In other work, fluctuations were counterbalanced by real-time computer normalization using a model provided by a trained artificial neural network (ANN) [32]. Although showing promise, this technique requires a large dataset to train the algorithm and ultimately reach the desired accuracy.

Here, we report a drone-mountable sensing platform using a graphene chemiresistor array fabricated integrated to a battery-powered μC development board and accessible with short- and long-range wireless communication systems. More importantly, we demonstrate the possibility to integrate graphene-based sensing technology into a drone-mountable platform for real-life applications. We propose a compact design and explore some of the main limitations, including environmental noise caused by temperature and humidity fluctuations. In a lab setting, the device reading accuracy is similar to the one provided by a state-of-the-art multi-meter (Model 2400, Keithley Instruments, Solon, OH, USA). Tests performed during flights and under rapidly changing environmental conditions show a significant increase in noise level. We were able to reduce signal fluctuations by about an order of magnitude when normalizing the signal against independent temperature and humidity readings from a commercial sensor also integrated into the sensing platform.

## 2. Materials and Methods

**Figure 1:**
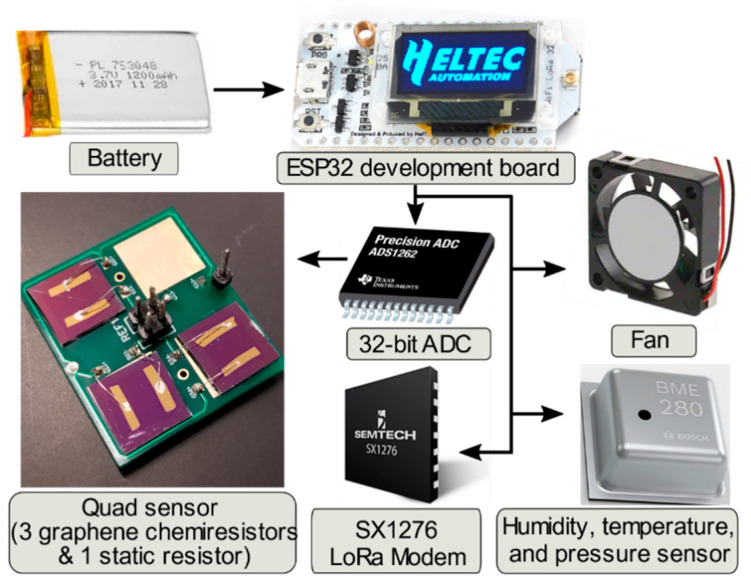
Diagram of the main functional components of the gas sensing platform. A battery powers the development board (ESP32 μC), which controls the graphene chemiresistor array via a 32-bit analog-to-digital converter (ADC). A fan, a long-range communication (LoRa) module and a commercial humidity/temperature/pressure sensing device are also accessible by the ESP32 μC using the I2C communication bus.

We design a compact and light-weight (185 g) sensing platform using commercially available μC devices and compatible components (see Figure 1). The functional component is an ESP32-based development board, which controls the general operations of the device. The sensor array contains three graphene chemiresistors, which consist of 0.7 × 0.7 cm CVD graphene layers placed onto a 1.0 × 1.0 cm Si/SiO_2_ substrate with parallel gold electrodes deposited on the surface. The chemiresistors are fabricated following the technique described in [17]. Although the board can accommodate a fourth graphene sample, in this experiment we use instead a commercial static resistor, which monitors the electronic stability of the system.

A high-accuracy 32-bit analog-to-digital converter (ADC) monitors real-time resistance of the four devices by measuring the voltage across four reference resistances directly mounted on the sensor board. Note that the ADS1262 ADC model used in this configuration could allow up to eight channels to be read simultaneously. The μC performs a circuit calculation using these voltages to retrieve the resistance of each three chemiresistors and the static resistor (details in Supplementary Materials). These values are sent in data packets to another remote μC device, referred here as the gateway, via Semtech’s LoRa protocol using a 915 MHz center-frequency. The gateway then unpacks the packets and sends the resistance digital values to either: (i) a computer via serial cable or (ii) a smartphone running a mobile application allowing data transfer over Bluetooth. The LoRa communication in our application has been tested to work consistently at distances up to 1 km from the operator while sending a steady stream of data. This range can be improved by using higher gain antennas. Components used in the device configuration, such as the μC, ADC converter, and fan, are commercially available; here we present an original design, assembly, and overall architecture involving these components to demonstrate a drone-mountable sensing platform.

A commercial temperature and humidity (TH) sensor (model BME280) monitors environmental fluctuations. Its readings are used to normalize the graphene sensors’ response as we demonstrate later. A comprehensive functional diagram including all device components can be found in Figure S3 (see Supplementary Materials). The information collected by all sensors comprised in the platform is registered every 210 ms (or at a rate of 4.8 Hz). However, the device performs a moving average to reduce noise, yielding a time resolution of ~1 s and matching the time resolution of the BME280 sensor.

**Figure 2:**
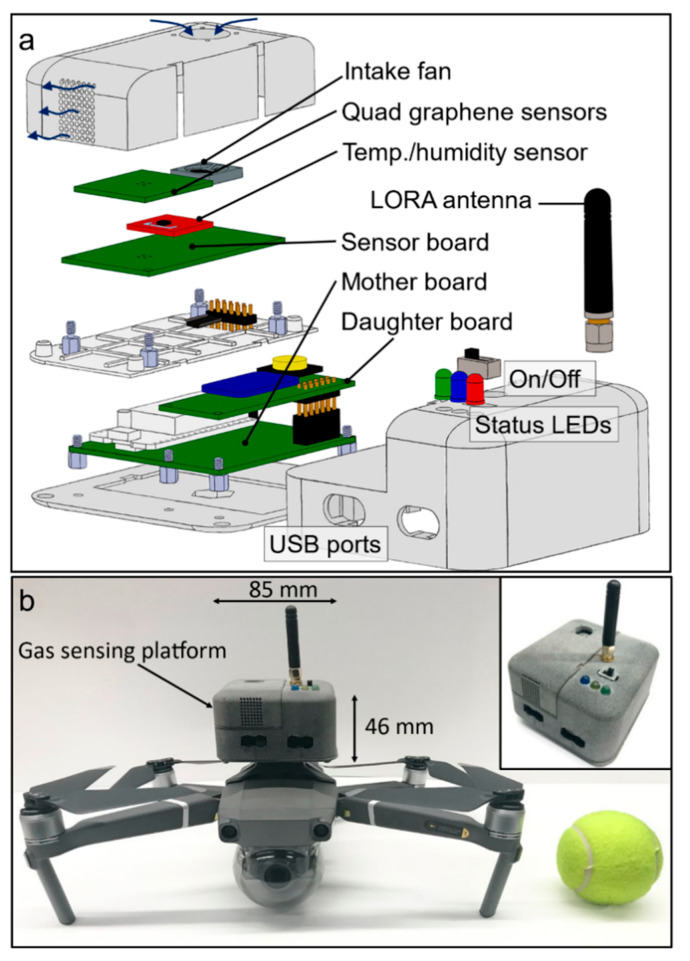
(**a**) Schematic of the gas sensing platform showing components and configuration. (**b**) Photo of the gas sensing device mounted on top of a drone, and unmounted in inset, with dimensions comparable to a tennis ball.

The sensing platform architecture is adapted to the drone size and shape (see Figure 2). The 3D printed casing has a length and width of ~85 mm and a height of 46 mm. An intake fan located at the top of the device allows air to flow across the sensing chamber, which contains both the quad sensor panel and TH sensor, and then exit by the exhaust located on the side of the casing. USB ports are used to charge the lithium-ion battery (right port) and access the ESP32 μC (left port) for firmware updates. PCB boards are stacked vertically to minimize lateral footprint. An adapter at the base of the casing ensures solid mounting of the device on the top of the drone. Note that the platform could not easily be located under the drone as it tends to obstruct optical sensors necessary for the drone’s advanced obstacle avoidance system.

**Figure 3:**
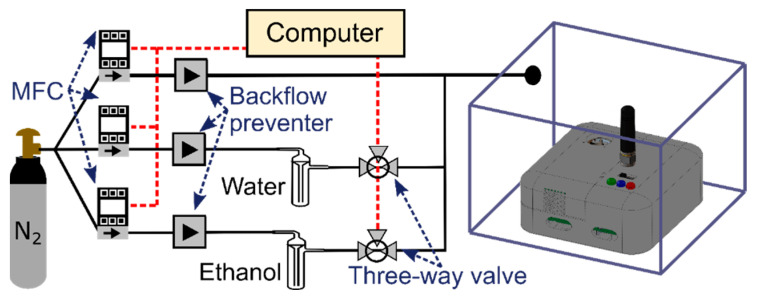
Schematic of the automated experimental setup used to characterize the sensor responsivity to different chemicals species (ethanol and water vapour) diluted in a N_2_ carrier gas. The sensing platform is located inside a plexiglass enclosure.

We demonstrate the practicality of the device by testing it under different experimental conditions: (1) in a laboratory setting, inside an enclosure under a controlled environment, (2) mounted on a drone during an indoor flight, and (3) outdoor while being exposed to wind and sun. In the laboratory, experiments are conducted with a standard configuration (see Figure 3) for evaluating the sensing platform’s response when exposed to precise concentrations and exposure times of two target analytes: ethanol and water vapour. Although graphene-based chemiresistors are not especially sensitive to ethanol [33], here we use this low-cost, easily accessible, and low-toxicity chemical to demonstrate the concept of a compact and portable sensing platform. Three mass flow controllers (MFCs) are used to control the concentration of gases supplied inside a plexiglass enclosure in which the sensing platform is placed. Two gas lines are connected to a bubbler to provide saturated vapours of ethanol and water. Computer-automated 3-way valves allow us to determine the precise exposure time to each analyte. Indoor flight tests are conducted inside a 10 m × 5 m × 4 m drone area, allowing us to investigate the effect of the drone movement on the sensors. We also explore the ability to detect humidity changes while flying the drone. In these experiments, the drone is initially placed at one end of the drone cage, and a commercial humidifier at the opposite end. We fly the drone within 1 m of the water vapour jet of the humidifier for the detection test. Finally, in the third experimental setting, the sensing platform was tested outdoor under rapidly fluctuating conditions due to wind and sun exposure.

## 3. Results and Discussion

### 3.1. Tests in a Laboratory Setting

**Figure 4:**
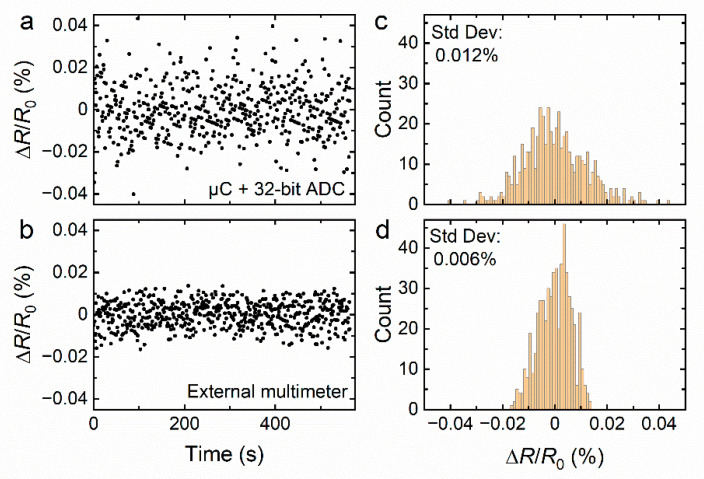
Resistance measurements of a graphene sensor under steady state conditions (**a**) measured with our sensing platform combining a μC and 32-bit ADC, and (**b**) extracted from the linear *I-V* characteristic curve obtained by an external multimeter (Keithley 2400) by keeping the voltage constant and reading the current. (**c**,**d**) show the statistical distribution of the time-dependent data presented in (**a**,**b**), respectively.

In a first experiment, the sensing platform is placed inside a plexiglass enclosure under steady conditions and we investigate the optimal sensitivity of the device by analyzing the noise characteristics with a series of continuous measurements. Figure 4a shows the corresponding data points collected by the sensing platform’s ESP32 μC and ADC components during a period of 575 s. Figure 4c shows that the distribution of data points is Gaussian-like and has a standard deviation of Δ*R/R*_0_ = 0.012%. The response Δ*R/R*_0_ provided by a sensor is defined as the change of resistance Δ*R* of the graphene sheet divided by its resistance *R*_0_ at the beginning of the experiment. We performed a similar series of measurements using a state-of-the-art sensor monitoring system. This system extracts the electrical resistance from the linear *I-V* characteristic curve obtained by a multimeter (Keithley 2400) by keeping the voltage constant and reading the current. Results shown in Figure 4b,d indicate a noise level that is only larger by a factor of two (Δ*R/R*_0_ = 0.006%). Considering that the standard laboratory data acquisition equipment is significantly more bulky and expensive than our compact sensing platform, we believe this comparison not only demonstrates the ability to use μC-based infrastructure for sensitive remote gas sensing, but also its potential to replace standard data acquisition equipment to save space and costs. All data acquisition in Figure 4 is performed at the common sampling rate of ~1 Hz.

**Figure 5:**
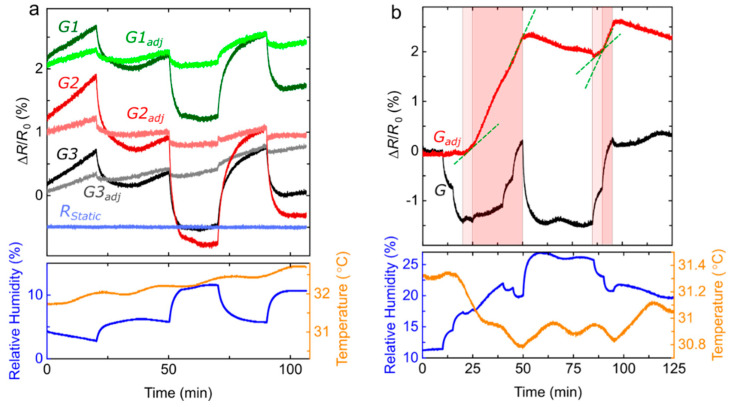
(**a**) The sensing platform inside an enclosure allows us to remotely monitor the signal (Δ*R*/*R*_0_) of three graphene sensors, *G1*, *G2*, and *G3*, one static resistance *R_Static_* (top panel, with a vertical offset of 1% between all readings), as well as the relative humidity and temperature provided by the TH sensor (bottom panel). As we introduce varying concentrations of water vapour inside the enclosure a correlation is observed between the *G*’s and the humidity. We use Equation (1) to adjust the graphene sensors’ response to a signal*, G_adj_*, more independent to fluctuations in humidity and temperature. (**b**) The experiment is repeated with one graphene sensor while introducing different concentrations of ethanol vapour (red regions) and water vapour (not indicated with background color but traceable from the relative humidity readings). The light red region corresponds to an ethanol vapour concentration of 7800 ppm and the darker red region to a higher concentration of 13,700 ppm.

In another experiment, also performed in the laboratory setting described in Figure 3, the sensing platform monitors devices’ resistance changes when we introduce analytes into the plexiglass enclosure. Figure 5a shows the recorded resistance changes of the three graphene sensors, *G1*, *G2*, and *G3*, and the static resistance, *R_Static_*, (top panel), as well as the recorded relative humidity and temperature provided by the TH sensor (bottom panel). In this experiment, water vapour is first introduced in the enclosure. We see a correlation between the humidity level (blue line) and the signal (Δ*R*/*R*_0_) provided by the three graphene sensors, where *R*_0_ is the resistance at time 0 s. The fact that *R_Static_* remains constant during the experiment demonstrates the stability of the electronic configuration and reliability of the resistance readings.

The signal *G* = Δ*R*/*R*_0_ of a graphene sensor depends on ambient humidity and temperature, which are varying parameters difficult to control in real-life applications. In practice, this can result in signal fluctuations reducing the sensor’s sensitivity to a targeted gas. Here, we independently measure humidity and temperature and use signal processing to account for the effects of these two environmental parameters on the sensor. We build a model, based on previous work [13,31], to calculate *G_mod_*, the projected signal derived from humidity and temperature readings only:

(1) $$G_{\mathrm{mod}}=a\Delta H_{abs}e^{\frac{E_{d}}{N_{A}k_{B}T}}+b{\left(T-273.15\right)}^{c}$$

where *T* is the temperature in K, $\Delta H_{abs}\left(T\right)$ is the change in absolute humidity, *N_A_ k_B_* (referred as the gas constant 8.314 J/mol K) is the Avogadro’s number multiplied by the Bolzmann constant, and *E_d_* is the energy for water desorption (4.64 × 10^4^ J/mol [34]). The variables *a*, *b*, and *c*, are determined for each individual graphene sensor, as the relationship between the signal and the parameters in Equation (1) depends on intrinsic graphene electrical properties, such as the doping level [34]. These three variables are determined by minimizing the adjusted signal ($G_{\mathrm{adj}}$ = $G-G_{\mathrm{mod}}$) during an experiment performed under rapidly fluctuating environmental conditions. We use the same values, listed in Table 1, in all other experiments to obtain the adjusted signal. Signal variations due to changes in humidity or temperature can then be significantly decreased, by about an order of magnitude (Figure 5a).

Figure 5b demonstrates the practicality of this normalization step to detect ethanol vapours. In this experiment, we trace the signal of a single graphene sensor while varying the concentrations of water and ethanol, both successively and simultaneously, inside the enclosure containing the sensing platform. The monitored relative humidity (blue line, bottom panel) indicates significant and random-like modulations, which are also reflected in the original signal *G*. After adjustment, most of these modulations are cancelled out and we are left with a signal perfectly correlated with the exposure times to ethanol. More strikingly, the slope of Δ*R*/*R*_0_ (green dashed lines) is changing accordingly to the ethanol concentration, which is equal to 7800 ppm in the light red region and 13,700 ppm in the darker red regions. Chemiresistors used in this work have a response time of ~20 min [10], which is defined as the time required for the sensor in a lab setting to display a signal Δ*R/R*_0_ reaching 90% the maximum signal associated with a constant analyte concentration. For real-life applications, other features, such as the maximum slope of the time-dependent signal, or a comparison between the responses provided by different types of sensors, could be monitored to reduce the time necessary to detect and identify chemicals [19]. Furthermore, a shorter response time and recovery time can be achieved by structural modifications of the sensing platform, e.g., by optimizing the chamber design [35], implementing a functionalization of the graphene surface [36], or using of a heterodyne sensing configuration [37]. The tests performed in a controlled laboratory setting show promises for the sensitive detection of specific gases in field applications under fluctuating environmental conditions. However, more external factors can potentially influence the signal when the sensor is mounted on a flying drone.

### 3.2. Indoor Flight Tests

**Figure 6:**
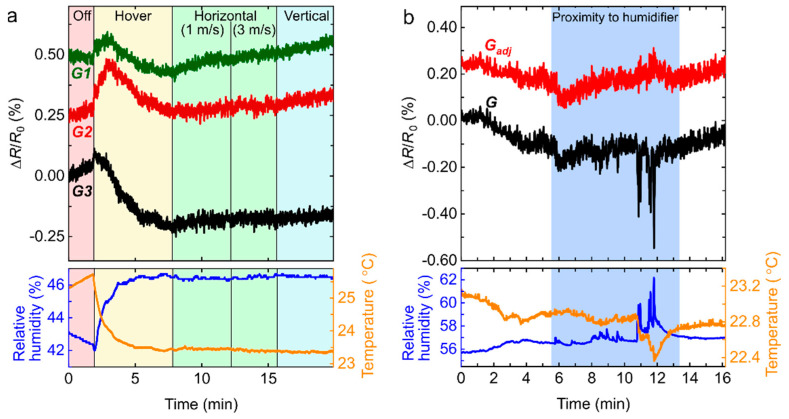
The sensing platform mounted on a flying drone measures and relays in real-time the changes in resistance of graphene sensors while also monitoring the relative humidity and temperature with a commercial sensor (bottom panel). (**a**) The changes in the sensing response as a function of drone motion is investigated by performing measurements when the drone is powered off and on the ground (red region), hovering (yellow region), moving horizontally at speeds of 1 m/s and 3 m/s (green regions), or moving vertically (blue region). (**b**) As the flying drone is approaching a humidifier, the signal *G* relayed by the graphene sensors (only one is shown here) features large irregular oscillations. Equation (1) and parameters listed in Table 1 allow us to calculate *G_adj_*, a signal independent of environmental variations such as humidity and temperature changes.

As a first step towards assessing the performance of the drone platform in real-life environments, we mounted the sensing platform on a drone while collecting data with the sensors on-board. Figure 6a shows the signal measured while the drone is in motion. After switching on the sensing platform electronics and before activating the drone propellers (still switched off), we observe a slow but gradual rise in temperature causing a drift in the signal of all sensors. When the drone is in flight mode and starts hovering 0.5 m above the floor, the signal initially oscillates significantly but, within a minute, tends towards a plateau due to the increased air flow contributing to regulating the temperature. The drone is then kept at a constant height and repetitively moved over an 8 m distance at different speeds, first at ~1 m/s and then ~3 m/s. No significant changes can be observed due to the motion of the drone in the horizontal direction. We draw the same conclusion when the drone is repetitively moved vertically from 0.5 m to 3.1 m during the last series of measurements for this test.

We also investigated the response of the graphene sensors to a sudden increase in humidity during in-flight tests by approaching the flying drone to a humidifier releasing a jet of water vapour (Figure 6b). Interestingly, the monitored humidity varies irregularly in time and the largest related signal is observed during the time period corresponding to 11 to 12 min. We attribute the irregular fluctuation to the local increase in humidity induced by the humidifier jet, which is periodically sampled by our sensing platform as the drone propellers mix up different volumes of air in a turbulent manner. By monitoring the humidity and temperature with a TH sensor, we then use Equation (1) and the parameters listed in Table 1 to calculate *G_adj_* and significantly reduce the effects of fluctuating humidity.

### 3.3. Outdoor Tests

**Figure 7:**
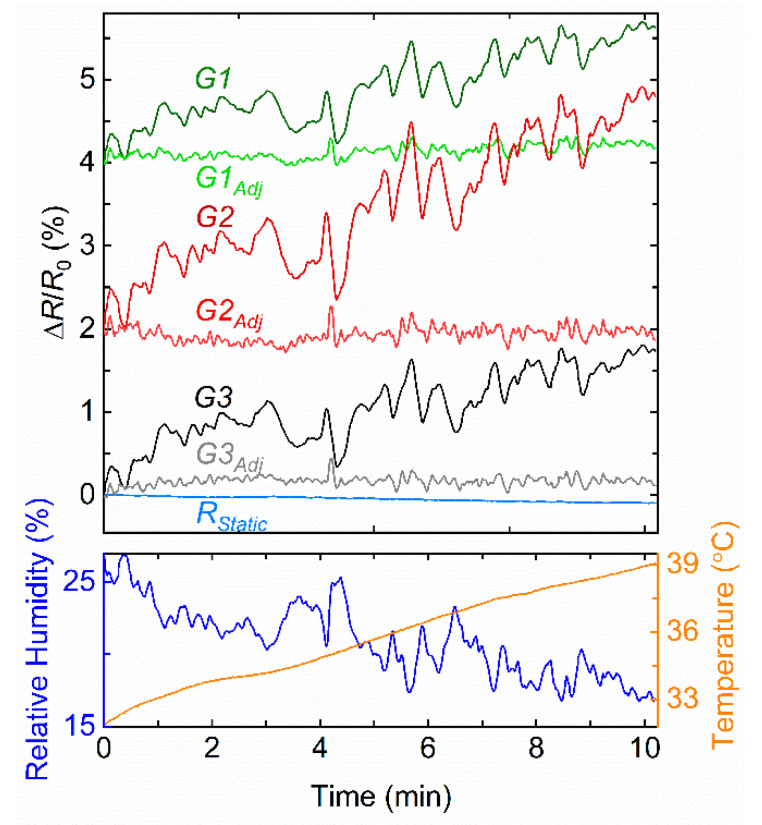
The sensing platform located outdoor allows us to remotely monitor the signal (Δ*R*/*R*_0_) of three graphene sensors, *G1, G2*, and *G3*, one static resistance *R_Static_* (top panel), as well as the ambient relative humidity and temperature provided by the TH sensor (bottom panel). Equation (1) is used to decrease the effects of the change in humidity and temperature on *G*, to obtain the adjusted signal *G_adj_*. A vertical offset of 2% for *G2/G2_adj_* and 4% for *G1/G1_adj_* is added for clarity.

In a final experiment, we investigate the response of the sensing platform when it is left outdoor (under direct sunlight at an ambient temperature of 24 °C, humidity 27%, and wind speed 11.3 km/h), while being exposed to fluctuating environmental conditions. The results are presented in Figure 7. After switching the device on, we immediately notice an increase in temperature due to the electronics warming up the device, but more importantly because of the direct sunlight exposure. This leads to a gradual increase in the graphene sensor signal, which simultaneously shows relatively large and fast fluctuations correlated to the varying relative humidity. We apply the normalization procedure based on Equation (1) to reduce the influence of these environmental factors and notice a significant decrease in signal fluctuation in the adjusted signal *G_adj_*. The standard deviation of the three signals from the graphene sensors (*G1*, *G2*, *G3*) decreases, on average, from 0.53% to 0.08%. In other words, the noise decreases by a factor of ~7. For the experiment shown in Figure 7, the commercial TH sensor is an HDC1080 (instead of the BME280 used in previous experiments) with a time response approaching 10 s. As a result, a 50-sample moving average is applied on the graphene sensor signals to optimize the signal synchronization between all sensors. We would expect to significantly improve noise reduction using a TH and graphene sensors having a perfectly matching temporal response.

## 4. Conclusions

We demonstrate the use of a microcontroller (μC) and related components to produce a lightweight, compact, and low-cost platform, which can be mounted on a drone for remote gas monitoring over distances of 1 km. Under controlled conditions, our platform can relay a signal *G (*Δ*R/R*_0_*)* with relatively low noise (0.06%), only two times higher than the one measured with standard, state-of-the-art laboratory equipment. When exposed to water and ethanol vapours, graphene sensors show a clear response. We build a model of the graphene response as a function of monitored changes in humidity and temperature, and use it to obtain an adjusted signal *G_adj_* independent of these two environmental parameters and distinctively revealing the presence of specific gases. We demonstrate this concept by exposing our sensors to fluctuating water vapour concentrations while detecting ethanol. Indoor flight tests show that the flying drone motion does not significantly affect the sensing response, especially when the platform temperature has reached an equilibrium. Finally, we demonstrate that fast-varying outdoor environmental conditions increase graphene-based chemiresistor noise. This poses a major obstacle to the deployment of sensing technologies in the field. However, cross-data analysis on data provided by a commercial temperature/humidity (TH) sensor and graphene sensors can be used in real-time to decrease unwanted signals created by environmental fluctuations. We demonstrate a noise reduction approaching an order of magnitude with room for further improvements, which can be achieved by perfectly matching the time response of the TH and graphene sensors. In our experiment, three graphene chemisensors fabricated with the same technique are tested simultaneously to demonstrate reproducibility. This compensation method for temperature and humidity is software-driven and can be easily applied to graphene-based sensor systems. In a lab-setting, we demonstrate that our platform can achieve a signal-to-noise comparable to the one obtained with high-quality commercial multimeters. Previous work has established correlations between air flow and the relative positioning of the sensors. The fan included in our device architecture helps sample the surrounding area but a more in-depth study of the placement and orientation of the air intake could still improve the overall performances of our gas sensing platform [38,39,40]. In future experiments, selected graphene-based sensors could be functionalized chemically or optically [33] to enhance sensitivity to specific gases and improve selectivity. A different sensing board design could also accommodate a larger number of sensors with graphene sheets of smaller area [41].

## Figures and Tables

**Table 1:** Fitting parameters used in Equation (1) to remove humidity and temperature dependence on signals from the three graphene devices in our sensing platform.

	*a* (×10^−9^)	*b* (×10^−4^)	*c* (×10^0^)
*G1*	4	1.5	2.8
*G2*	7.5	1.6	2.85
*G3*	4.9	1.65	2.7

## Data Availability

The data supporting the findings of this study are available within the article and its supplementary materials.

## Supplementary Materials

The following are available online at https://www.mdpi.com/article/10.3390/s22062383/s1, Figure S1: High-level circuit schematic of resistance monitoring module of the graphene sample (a) Sensor board, (b) Analog to digital converter (ADC), (c) ESP32 microcontroller, Figure S2: Schematic of the circuit and sample calculation to retrieve the resistance of the graphene chemiresitor *G*1. *V*_0_ is provided by an external battery and voltage regulator, *R*_G1_ is the actual resistance of the graphene sensor *G1*, *R1_Ref_* is a 1 kΩ commercial resistor and *V*_1_ is the voltage read by the ADC. The same technique is applied to monitor the resistance of other graphene samples on-board the sensing platform as well as the static resistor, Figure S3: Complete functional schematic of the sensing platform.
