# Supplementary material for: Drone-Mountable Gas Sensing Platform Using Graphene Chemiresistors for Remote In-Field Monitoring
Source: Sensors (Basel). 2022 Mar 19;22(6):2383. doi: 10.3390/s22062383 (PMC8954879; doi:10.3390/s22062383)
Supplement: Supplementary file 1 [file sensors-22-02383-s001.zip › sensors-1603783-supplementary.pdf]

# Supplementary Materials

*Article*

# Drone-Mountable Gas Sensing Platform Using Graphene Chemiresistors for Remote In-Field Monitoring

**Jaewoo Park, Franklyn Jumu, Justin Power, Maxime Richard, Yomna Elsahli, Mohamad Ali Jarkas, Andy Ruan, Adina Luican-Mayer and Jean-Michel Ménard ***

Department of Physics, University of Ottawa, Ottawa, ON K1N 6N5, Canada; jpark4@uottawa.ca (J.P.); fjumu014@uottawa.ca (F.J.); jpowe074@uottawa.ca (J.P.); mrich154@uottawa.ca (M.R.); yelsa078@uottawa.ca (Y.E.); mjark066@uottawa.ca (M.A.J.); a2ruan@uwaterloo.ca (A.R.); luican-mayer@uottawa.ca (A.L.-M.)

* Correspondence: jean-michel.menard@uottawa.ca

**Monitoring the resistance of four devices:**

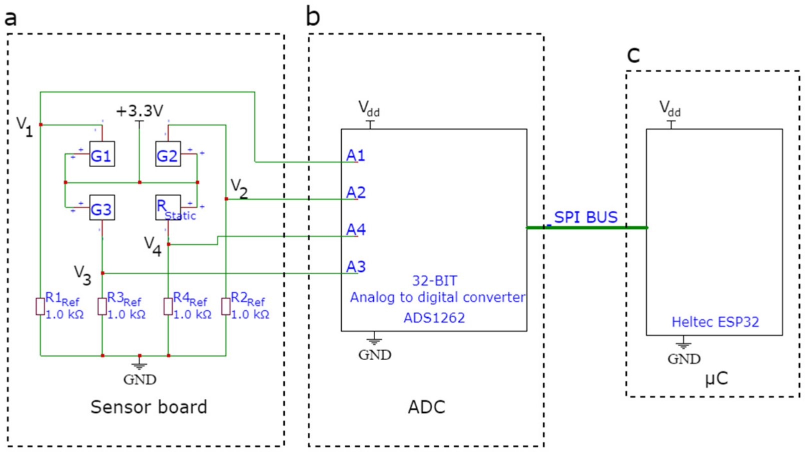


**Figure S1.** High-level circuit schematic of resistance monitoring module of the graphene sample (a) Sensor board, (b) Analog to digital converter (ADC), (c) ESP32 microcontroller.

Our sensing platform contains three graphene chemiresistors (G1, G2, G3); To detect the presence of a chemical, one monitors the resistance of these graphene samples. Our platform also contains a commercial resistor $R_{Static}$, which resistance is also monitored to ensure the system's electronic stability. The architecture used to monitor the resistance of each graphene samples is composed of three main components. The first, the sensor board, can house up to four sensors which are connected in series to a 1 kΩ reference resistances and terminated at a common ground. A 3.3 DC voltage ($V_0$) is provided to the sensor board via the external battery and voltage regulator. The regulator ensures that the input voltage to the sensor board is steady and resistant to voltage fluctuations that may compromise the final resistance reading.

The second component, the analog to digital converter (ADC), takes an analog voltage reading over each reference resistor in succession at points $V_1$, $V_2$, $V_3$, and $V_4$. Due to the multiplexing capability of the ADC, each voltage reading can be considered independent from the next. The ADC is then able to convert each analog voltage reading into a digital value that can be communicated through SPI data bus to the final component in our architecture, an ESP32 microcontroller (µC). The µC uses the digital voltage values and performs a simple ohms law calculation to recover the ohmic resistances (see equation in Fig. S2). The process of obtaining these resistances is performed on a continuous basis. We can program the µC to perform a moving average to reduce noise. The calculated

averaged values of each resistance are then transmitted in data packets by wireless communication via the Semtech's LoRa protocol.

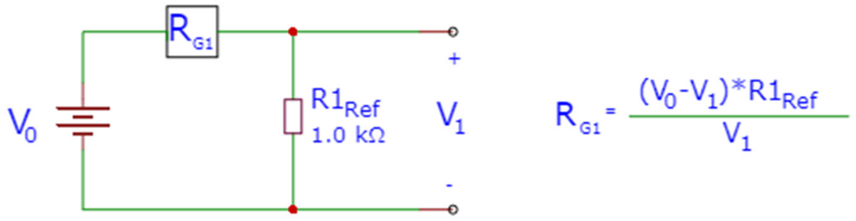


**Figure S2.** Schematic of the circuit and sample calculation to retrieve the resistance of the graphene chemiresitor G1. $V_0$ is provided by an external battery and voltage regulator, $R_{G1}$ is the actual resistance of the graphene sensor G1, $R1_{Ref}$ is a 1 kΩ commercial resistor and $V_1$ is the voltage read by the ADC. The same technique is applied to monitor the resistance of other graphene samples onboard the sensing platform as well as the static resistor.

### Hardware architecture – devices and components:

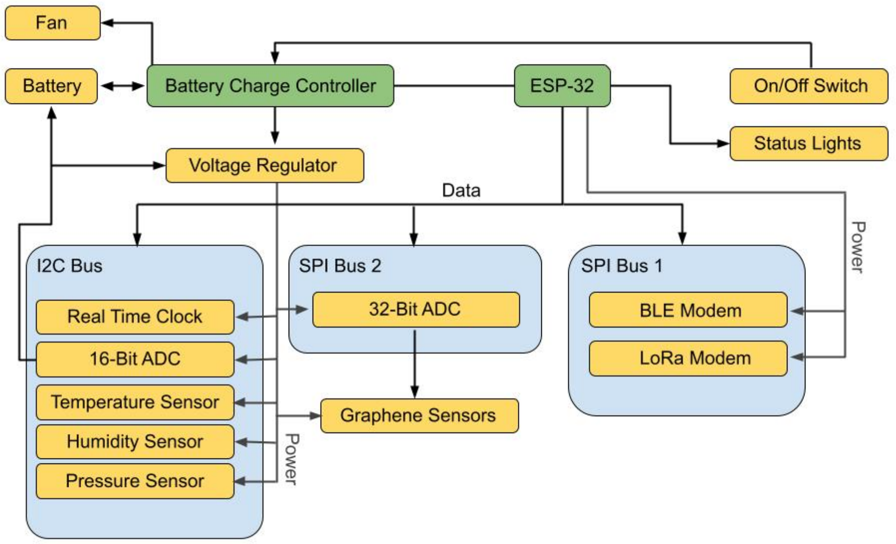


**Figure S3.** Complete functional schematic of the sensing platform.
